# The Mitochondrial Kinase PINK1 in Diabetic Kidney Disease

**DOI:** 10.3390/ijms22041525

**Published:** 2021-02-03

**Authors:** Chunling Huang, Ji Bian, Qinghua Cao, Xin-Ming Chen, Carol A. Pollock

**Affiliations:** Kolling Institute, Sydney Medical School, Royal North Shore Hospital, University of Sydney, St. Leonards, NSW 2065, Australia; jbia3972@uni.sydney.edu.au (J.B.); qinghua.cao@sydney.edu.au (Q.C.); xin-ming.chen@sydney.edu.au (X.-M.C.)

**Keywords:** PINK1, diabetic kidney disease, mitochondria, mitochondria quality control, mitophagy

## Abstract

Mitochondria are critical organelles that play a key role in cellular metabolism, survival, and homeostasis. Mitochondrial dysfunction has been implicated in the pathogenesis of diabetic kidney disease. The function of mitochondria is critically regulated by several mitochondrial protein kinases, including the phosphatase and tensin homolog (PTEN)-induced kinase 1 (PINK1). The focus of PINK1 research has been centered on neuronal diseases. Recent studies have revealed a close link between PINK1 and many other diseases including kidney diseases. This review will provide a concise summary of PINK1 and its regulation of mitochondrial function in health and disease. The physiological role of PINK1 in the major cells involved in diabetic kidney disease including proximal tubular cells and podocytes will also be summarized. Collectively, these studies suggested that targeting PINK1 may offer a promising alternative for the treatment of diabetic kidney disease.

## 1. Introduction

Mitochondria, the power generators of the cell, perform a wide range of cellular functions [1]. Maintenance of mitochondrial function and turnover is critical to cellular metabolism, survival, and homeostasis. Mitochondrial damage and dysregulation of turnover lead to metabolic dysfunction, oxidative stress, inflammation, and cell death, all of which are involved in the pathogenesis of diabetic kidney disease (DKD) [2]. As a result, mitochondrial dysfunction has been recognized as a major contributor to the development and progression of diabetic kidney disease [3]. Preservation of mitochondrial homeostasis will theoretically prevent kidney damage and maintain normal kidney function. Studies have shown that mitochondrial function is mainly regulated by mitochondrial protein kinases [4]. Activation of those mitochondrial protein kinases has emerged as an important molecular mechanism mediating the mitochondrial response to metabolic stresses in DKD.

The phosphatase and tensin homolog (PTEN)-induced kinase 1 (PINK1) has received increasing attention as an important mitochondria-targeted kinase. Shortly after PINK1 was identified as a Parkinson’s disease-associated gene in 2004, research focusing on the biological roles of PINK1 have mainly cantered on neuronal diseases [5]. Recent studies have demonstrated the close association between PINK1 and many other diseases including cancer, diabetes, and kidney diseases [6]. This review will provide a concise overview of PINK1 and explore its cellular function in healthy and damaged mitochondria as well as its physiological role in kidney cells primarily involved in the initiation and progression of DKD.

## 2. Overview of PINK1

### 2.1. Expression and Characteristics of PINK1

The mitochondrial kinase PINK1 was identified and named in 2001 when it was recognised as being induced by the tumour suppressor gene PTEN in endometrial cancer cells [7]. PINK1, encoded by the PARK6 gene, consists of eight exons, which encode a 581 amino acid (aa) protein with N-terminal mitochondrial targeting sequences (MTS), a transmembrane domain (TMD), followed by a serine/threonine kinase domain, and a C-terminal putatively regulatory domain. Like most mitochondrial proteins, PINK1 polypeptide is encoded in the nucleus, synthesized at cytosolic ribosomes as a precursor, and transported into mitochondria.

Under healthy and steady-state conditions, PINK1 is imported into the mitochondria by a positively charged N-terminal MTS, via the presequence pathway, which is involved in the targeting of most matrix-localized proteins and some mitochondrial inner membrane (MIM)- and intra-membrane space (IMS)-localized proteins [8]. Through this cleavable N-terminal signal sequence, the precursor proteins are first recognized by cytosol-exposed Tom receptors (Tom20, Tom22, Tom70) of the mitochondrial outer membrane (MOM), guided into the Tom40 channel, and transferred to the Tim23 complex in the MIM. The proteins reach the mitochondrial inner membrane through the Tim23 channel, depending on mitochondrial transmembrane potential, the presequence translocase-associated motor (PAM), and the ATP-dependent mitochondrial heat-shock protein 70 [8].

A series of proteolytic cleavage events are involved in the PINK1 importing process. Whilst it spans the mitochondrial inner membrane, the N-terminal MTS domain reaches the matrix and is cleaved by the matrix-localized protease (MPP), resulting in a 60-kDa intermediate form (cleavage between aa 34 and 35) [9]. The mitochondrial inner membrane protease, presenilin-associated rhomboid-like protease (PARL), then catalyses a second cleavage between amino acids Ala103 and Phe104 within the PINK1 sequence, generating the 52-kDa processed PINK1 [10,11,12,13]. In addition, two other ATP-dependent mitochondrial proteases, matrix-AAA (m-AAA) and caseinolytic mitochondrial matrix peptidase have been reported to be involved in PINK1 cleavage [9]. However, the exact functions and the precise cleavage sites of these proteases need to be clarified. After cleavage by the different proteases, the mature and 52-kDa processed PINK1 is released into the cytosol, where its likely stabilized through the formation of complexes with different proteins or degraded by proteasomes [14]. The proteins that interact with PINK1 and regulate its stability include chaperone proteins such as Hsp90/Cdc37, members of Bcl-2–associated athanogene proteins such as BAG2, BAG5, and BAG6 [15,16,17,18] as well as the component of sorting and assembly machinery (Sam50) [19]. The degradation of 52-kDa PINK1 is primarily regulated by the ubiquitin/proteasome pathway via specific ubiquitination at Lys-137 (K137), but not by an N-end rule mechanism, as previously proposed [20,21]. Recently, a new proteasome degradation pathway for PINK1 has been identified, which relies on the interaction with the endoplasmic reticulum-associated degradation machinery, including valosin-containing protein (VCP), the E3 ligases gp78, and HRD [22]. As a result, in healthy mitochondria, PINK1 is constitutively imported, cleaved, and degraded, which leads to the low level of PINK1 in an inactive state and prevention of the PINK1 kinase activation.

### 2.2. The Function of PINK1 in Mitochondria

**Figure 1:**
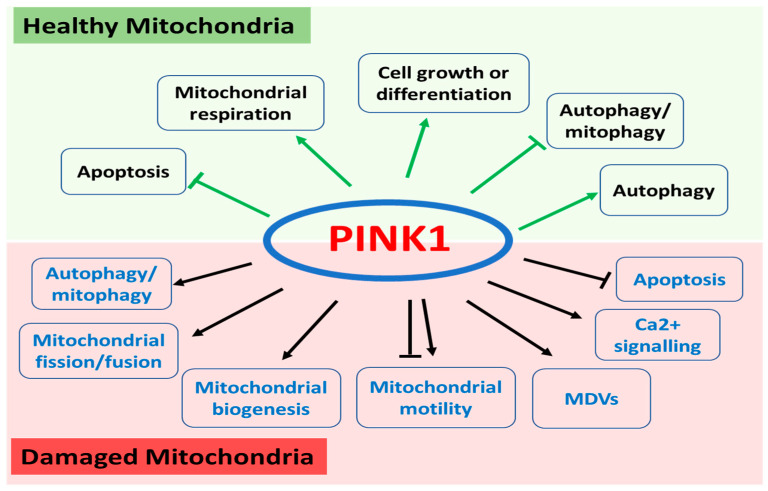
Functions of PINK1 in healthy and damaged mitochondria. In healthy mitochondria, PINK1 regulates apoptosis, mitochondrial respiration, cell growth or differentiation, and autophagy/mitophagy. In damaged mitochondrial, PINK1 plays a key role in autophagy/mitophagy, mitochondrial fission and fusion, mitochondrial biogenesis, mitochondrial motility, and mitochondria-derived vesicles (MDVs) as well as calcium signalling and apoptosis.

PINK1 is ubiquitously expressed throughout the body and localized in both mitochondria and cytosol. As one of the most diverse human protein kinases, PINK1 regulates a wide range of mitochondrial and cytosolic pathways through various substrates depending on its subcellular localization and the health status of the mitochondria (Figure 1).

#### 2.2.1. The Role of PINK1 in Healthy Mitochondria

In healthy mitochondria, recent studies have revealed that the N-terminal cleaved PINK1 released into the cytosol has distinct cellular functions working through different downstream substrates [14]. N-terminally truncated, cytosolic PINK1 protects against oxidative stress-induced apoptosis by phosphorylating the tumour necrosis factor receptor-associated protein 1 (TRAP1)/heat shock protein 75 (Hsp75), a member of the HSP90 family of the mitochondrial chaperone [23] and serine protease high-temperature-requirement protein A2 (HtrA2) [24]. PINK1 regulates mitochondrial respiration through the phosphorylation of complex I subunit, NADH dehydrogenase ubiquinone 1 alpha subcomplex 10 (NdufA10) [25]. Moreover, cytosolic PINK1 promotes neuronal growth and differentiation through activation of the mammalian target of rapamycin complex 2 (mTORC2)/Akt [26] as well as phosphorylation of PKA/p47 signalling pathways [27]. Interestingly, cytosolic PINK1 has been reported to suppress autophagy/mitophagy through modulation of mitochondrial fission [28] and PKA-mediated LC3 phosphorylation [29]. By directly binding to cytosolic Parkin, cytosolic PINK1 prevents the translocation of Parkin to the outer mitochondria and attenuates subsequent valinomycin-dependent mitophagy [30]. Alternatively, together with Parkin and cytosolic protein DJ-1 (PPD), forming a functional E3 ligase complex, PINK1 promotes ubiquitination and proteasomal degradation of misfolded Parkin substrates including Parkin itself and synphilin-1 to prevent initiation of mitophagy [31]. In contrast, the study by Gao et al. has identified the short form of cytosolic PINK1 as a major mediator of aggresome formation during proteasomal stress. The phosphorylation of the ubiquitin-binding protein SQSTM1/P62 (sequestosome 1) at Ser28 by PINK1 is required for efficient aggresome formation, indicating a major role of PINK1 in autophagic degradation [32].

#### 2.2.2. Activated PINK1 in Damaged Mitochondria

In response to mitochondrial damage, loss of mitochondrial membrane potential leads to the stabilization of PINK1 on the mitochondrial outer membrane. The accumulated full-length PINK1 on the MOM becomes dimerized, which subsequently triggers the autophosphorylation and activation of the kinase [33,34]. Activated PINK1 then functions as a major mitochondrial quality control protein in regulating mitochondrial homeostasis including mitophagy, fission, and fusion as well as biogenesis through the recruitment and phosphorylation of multiple substrates [6].

Mitophagy is the selective degradation of defective mitochondria by autophagy following damage or stress, which plays an important role in maintaining mitochondrial fitness. During the process of mitophagy, activated PINK1 triggers a cascade of events through different substrates. Firstly, PINK1 becomes activated through autophosphorylation at Ser228, Ser402, Thr257, Thr313, of which the first two affect Parkin and subsequent ubiquitin phosphorylation [33]. Parkin, an E3 Ubiquitin ligase, is one of the best-characterized downstream substrates of PINK1, and the PINK1/Parkin pathway is recognised as the major mediator of mitophagy. Stabilized PINK1 phosphorylates Parkin on Ser65 residue of its N-terminal ubiquitin-like domain and induces recruitment of Parkin to damaged mitochondria [35]. PINK1 also phosphorylates ubiquitin at residue Ser65, which led to the activation of Parkin E3 ligase activity [36]. Together, these actions provide a positive-feedback loop to activate the E3 ligase activity of Parkin, allowing it to ubiquitinate proteins located at the MOM interface and initiate mitophagy. In addition, PINK1 also actives several other ubiquitin E3 ligases to mediate Parkin-independent mitophagy, which include Siah E3 Ubiquitin Protein Ligase 1 (SIAH1) [37], Ariadne RBR E3 Ubiquitin Protein Ligase 1 (ARIH1) [38], and mitochondrial ubiquitin ligase 1 (MUL1) [39]. Phosphorylated PINK1 recruits and activates Parkin or other E3 ligases to ubiquitinate mitochondrial proteins, which then promotes the recruitment of mitophagy adapter proteins (i.e., nuclear dot protein 52 and optineurin) and facilitates clearance of the damaged mitochondria through ubiquitin-mediated mitophagy [40].

PINK1 has been shown to interact with components of the mitochondrial fission and fusion machinery, reflecting its role in mitochondrial dynamics [41]. Mitochondrial fission is required for mitophagy by generating small mitochondrial particles to be engulfed by the autophagosome. Dynamin-related protein 1 (Drp1) is a major regulator of mitochondrial fission. PINK1 promotes Drp1-dependent mitochondrial fission through the recruitment of Drp1 to mitochondria together with Parkin during mitophagy [42]. In addition, PINK1 acts as a powerful fission activator to promote mitochondria fission through indirectly regulating Drp1 activity through the A-kinase anchoring protein 1 (AKAP1)– protein kinase A (PKA) axis, which is independent of Parkin activity and calcium. A recent study confirmed that PINK1 can directly phosphorylate Drp1 at the Ser616 site, providing a novel mechanism for PINK1 in regulating mitochondrial fission [43,44]. Mitochondrial fusion is crucial in maintaining a healthy mitochondrial population by redistributing mitochondrial content to prevent the accumulation of damaged components. The mitochondrial fusion of the MOM is regulated by mitofusin 1 (Mfn1) and mitofusin 2 (Mfn2). Mfn1 and Mfn2 have been identified as novel ubiquitination substrates during mitophagy, which, as stated above, is dependent on PINK1 and Parkin [45]. PINK1 mediates the ubiquitination and proteasomal degradation of Mfn1/2 in a Parkin and p97-dependent manner to inhibit refusion of damaged mitochondria from healthy mitochondria and facilitate removal of impaired mitochondria [46]. Rocha et al. have identified and validated three PINK1 phosphorylation sites on human Mfn2 (Ser378, Thr111, Ser442) using mass spectrometry [47]. Phosphorylation of Mfn2 by PINK1 at Ser378 modulates mitochondrial fusion, whilst phosphorylation of Mfn2 by PINK1 at Thr 111 and Ser 442 serves as mitochondrial receptors for Parkin to promote mitophagy [47,48]. However, Mfn2 also acts as a mitochondria-endoplasmic reticulum (ER) tether to exert its antagonistic effect on mitophagy [49], which is independent of mitochondrial fusion. The phosphorubiquitination and degradation of Mfn2 mediated by PINK1/Parkin, disrupt mitochondria-ER contacts and releases ER from mitochondria to drive mitophagy, indicating a critical role of ER-mitochondria contacts for mitophagy [49].

Mitochondrial biogenesis, a process to increase mitochondrial mass, is an important part of the mitochondrial network. PINK1 has also been shown to be involved in mitochondrial biogenesis. PINK1 can directly influence mitochondrial DNA synthesis, which is necessary for mitochondrial biogenesis [50]. PINK1 kinase activity is required for Parkin-mediated ubiquitination and degradation of the Parkin interacting substrate (PARIS), a KRAB and zinc finger protein, via direct phosphorylation at serine residues 322 and 613 [51]. PINK1 depletion leads to accumulation of PARIS and repression of the transcriptional coactivator, peroxisome proliferator-activated receptor-gamma coactivator-1-alpha (PGC-1α), a master regulator of mitochondrial biogenesis, thereby inhibiting mitochondrial biogenesis [51]. The role of PINK1 in mitochondrial biogenesis via the PARIS/PGC-1α axis is further confirmed in Drosophila models [52].

There are a number of additional PINK1 substrates reported to be involved in mitochondrial quality control. Miro, a mitochondrial Rho GTPase at the mitochondrial outer membrane, is phosphorylated by PINK1 not only on S156 but also on T298/299. The status of Miro phosphorylation regulates mitochondrial motility through modulation of Parkin levels. PINK1-dependent phosphorylation of Miro on Ser156 recruits Parkin onto mitochondria and leads to Miro proteasomal degradation and inhibition of mitochondrial motility, while phosphorylation of Miro on T298/T299 exerts the opposite effect [53,54]. It is likely that substrate-driven activation through Miro forms another mode for Parkin activation in addition to PINK1 phosphorylation of ubiquitin or Parkin [53]. A phosphoproteomic screening study revealed that Rab GTPases including Rab8A, Rab8B, and Rab13 have been identified as novel downstream targets of PINK1. Activated PINK1 indirectly phosphorylates Rab GTPases Rab8A at serine 111, which negatively regulates leucine-rich repeat kinase 2 (LRRK2)-mediated phosphorylation of Rab8A at Thr72 [55,56]. Inhibition of LRRK2 kinase activity restored defective PINK1 dependent mitophagy in Parkinson’s disease, indicating a link between PINK1 and LRRK2 [56,57]. It is worth noting that PINK1 is also involved in the formation of mitochondria-derived vesicles (MDVs), which is an alternative pathway for mitochondria degradation and different from canonical mitophagy [58]. The stress-induced PINK1/Parkin-dependent MDVs selectively transport oxidized or damaged mitochondrial content to the late endosome/lysosome for degradation, which is mediated through syntaxin-17 [58].

In addition to its role in mitochondrial quality control, PINK1 plays a critical role in other cellular functions. For instance, PINK1 may promote autophagy through direct interaction with Beclin1, a crucial pro-autophagic protein [59]. PINK1 kinase activity is required for mitochondrial Ca^2+^ regulation. PINK1 directly regulates mitochondrial calcium efflux via the modulation of mitochondrial Na^+^/Ca^2+^ exchanger [60]. A mass spectrometry-based interactomics screen study identified the leucine zipper-EF-hand containing transmembrane protein 1 (LETM1), a mitochondrial inner membrane protein, as the potential PINK1 interacting proteins [61]. PINK1 phosphorylates LETM1 at Thr192 to mediate mitochondrial Ca^2+^ transport [61]. Moreover, PINK1 can protect against carbonyl cyanide m-chlorophenylhydrazine (CCCP)-induced apoptotic cell death through the phosphorylation of the anti-apoptotic protein Bcl-xL at serine 62, a member of the Bcl-2 family [62].

Collectively, it appears that PINK1 regulates mitochondrial quality control to maintain energy homeostasis including mitophagy (such as Parkin, ubiquitin, E3 ligases), mitochondrial dynamics (via Mfn1/Mfn2, Drp1), mitochondrial biogenesis (via PARIS), mitochondrial motility (via Miro) and MDVs (via syntaxin-17) as well as autophagy machinery (via Beclin 1), calcium signalling (via Na^+^/Ca^2+^ exchanger, LETM1), and apoptosis (via Bcl-xL).

Hence, the different isoforms of PINK1 are located in different subcellular pools, which exert multifunctional activities through various downstream substrates (Table 1). Further identification of substrates of PINK1 will provide a better understanding of its cellular functions.

## 3. Physiological Role of PINK1 in DKD

It is increasingly recognized that mitochondrial dysfunction has been implicated in the pathogenesis of DKD. PINK1 is present in the major cells involved in the development of DKD, including proximal tubular cells and podocytes (Table 2). Hence, targeting mitochondria to restore mitochondrial function may provide promising therapies for ameliorating DKD.

### 3.1. PINK1 in Tubular Epithelial Cells

Proximal tubule epithelial cells, the most prominent cell type in the renal cortex, play a role in the reabsorption of fluid, glucose, amino acids, and other substances filtered by the glomerulus. Proximal tubule cells are rich in mitochondria with high energy demand and are, therefore, vulnerable to mitochondrial dysfunction [72]. Growing evidence suggests that proximal tubule injury is closely related to renal function abnormalities and is increasingly recognized as a key mediator in the initiation and progression of DKD [73,74].

The role of PINK1 in tubular cells under diabetic conditions is emerging, with recent evidence suggesting that PINK1 mediated mitochondrial quality control, especially dysregulated mitophagy, is involved in diabetic tubular injury. Zhan et al. reported that hyperglycaemia inhibited the expression of PINK1, punctate LC3 (microtubule-associated protein 1A/1B-light chain 3), and mitochondrial profusion protein Mfn2, but increased the expression of mitochondrial fission protein Drp1 and Fis1 in renal proximal tubular cells under high-glucose conditions and in streptozotocin (STZ)-induced type 1 diabetic mice [63]. The abnormal changes indicated impaired mitochondrial quality control with increased mitochondrial fragmentation and reduced mitophagy, which was attenuated by inhibition of Myo-inositol oxygenase (MIOX), a tubular-specific enzyme, with D-glucarate [63]. Similarly, PINK1 mediated mitophagy was also inhibited in tubular cells of 24-week old db/db mice, a type 2 diabetic mouse model. The defective mitophagy in diabetic mice was characterized by decreased PINK1 transcription and Parkin phosphorylation, which was accompanied by aberrant mitochondrial dynamics with Drp1 activation and Mfn2 suppression. MitoQ treatment attenuated renal injury and tubulointerstitial fibrosis in DKD through the restoration of mitophagy in diabetic kidneys, which is mediated via the Nrf2/PINK pathway [64]. In contrast, the increased expression of PINK1 was reported in four-week STZ-induced diabetic rats together with increased expression of Drp1. Diabetes-induced renal mitochondrial dysfunction may be associated with the loss of renal calpain 10, a mitochondrial and cytosolic Ca^2+^-regulated cysteine protease [65]. A study by Liu et al. demonstrated that PINK1/Parkin mediated mitophagy, as evidenced by the upregulated levels of PINK1 and Parkin, was activated in the kidneys of 21-week old db/db mice [66]. These findings were further confirmed by the same group in their recent study [67]. The authors argued that the discrepancy in the expression level of PINK1 and the state of the mitophagy in DKD is likely due to the animal model selection, the experimental cycle, and blood glucose levels since the level of mitophagy changes along with the progression of DKD [67]. Hence, further investigation using PINK1 gene knockout animal models are needed to better understand the exact role of PINK1 in DKD.

### 3.2. PINK1 in Podocytes

Podocytes are highly specialized cells with a critical role in maintaining the glomerular filtration barrier. Podocyte damage or dysfunction contributes to proteinuria, which is one of the earliest features observed in DKD [75]. Although cellular mitochondrial content in podocytes is lower than tubular epithelial cells, podocyte mitochondrial dysfunction caused by defective mitophagy is involved in diabetic glomerular lesions, indicating the importance of podocytes in DKD [76]. Thus, maintaining the homeostasis of podocyte cells is crucial to the preservation of glomerular function.

Incubation of mouse podocytes in high glucose (HG) medium for 72 h inhibited mitophagy activity as evidenced by reduced expression of PINK1, which was accompanied by decreased transcriptional activity of forkhead-box class O1 (FoxO1) and increased podocyte apoptosis [68]. Overexpression of FoxO1 reduced podocyte apoptosis and decreased podocyte cell damage through upregulation of PINK1 expression in HG-treated podocytes and glomeruli of diabetic mice, indicating the interaction between PINK1 and FoxO1 [68]. The same group further confirmed that hyperglycaemia inhibited mitophagy in podocytes, which resulted in aberrant mitochondrial morphology and mitochondrial dysfunction in cultured mouse podocyte cells (CIMPs) and STZ-induced diabetic mice [69]. Through studying the promoter activity, it was confirmed that PINK1 is a direct downstream target of FoxO1, which acts mainly through the PINK1-binding site [69]. The upregulation of FoxO1 protected against high glucose induce mitochondrial dysfunction and podocyte injury in both in vitro and in vivo diabetic models, which was mediated through activating PINK1/Parkin-dependent mitophagy [69]. Zhou and colleagues also reported remarkable decreases in the expression of PINK1, PARK2, PGC-1α, and Sirt1, indicating that mitophagy and mitochondrial biogenesis were impaired in DKD [70]. The administration of progranulin (PGRN), a secreted glycoprotein, prevented the progression of DKD by selective elimination of dysfunctional mitochondria through mitophagy and induction of mitochondrial biogenesis to maintain mitochondrial homeostasis in podocytes [70]. Diabetic dyslipidemia is one of the major risk factors for the development and progression of DKD. Interestingly, palmitic acid (PA) induced increased expression of PINK1 and Parkin in podocytes, indicating that PINK1/Parkin-mediated mitophagy was activated in lipid-induced lipotoxicity, which was confirmed in the rat model of HFD-induced obesity [71]. Parkin knockdown inhibited mitophagy in podocytes, which further enhanced PA-induced mitochondrial damage, mitoROS production, and podocyte apoptosis, suggesting that PINK1/Parkin-mediated mitophagy exerts protective function against hyperlipidemia in DKD [71].

Although the level of PINK1/Parkin-dependent mitophagy, as indicated by the expression level of PINK1, varies in different cell and animal models, it is widely accepted that PINK1-mediated mitophagy plays a protective role in DKD. Hence, activation of PINK1 activity to restore mitophagy may be of benefit in DKD.

## 4. Pharmacological Activation of PINK1

The PINK1 kinase activity can be increased directly by small molecule activators. The ATP analog N6-furfuryl ATP (kinetin triphosphate, KTP) was the first to be identified as a direct small molecular activator of PINK1 in cells, which enhances the PINK1 dependent phosphorylation and recruitment of Parkin to depolarized mitochondria to initiate PINK1-mediated mitophagy [77]. However, KTP, an ATP neo-substrate can only activate PINK1 in the presence of a mitochondria-depolarizing agent such as CCCP, which limits its application. Subsequently, Osgerby et al. employed the powerful ProTide phosphate prodrug technology to develop nucleoside-based molecules and demonstrated that kinetin riboside (KR) and its ProTide can enhance PINK1 activity, which is independent of mitochondrial depolarization [78]. Further in vivo studies to validate the activities of those compounds are warranted.

As PINK1 can be activated by the loss of mitochondrial membrane potential, another approach to indirectly increase the kinase activity of PINK1 could be to depolarise the mitochondrial membrane potential. This could be achieved using the proton ionophore, CCCP, carbonyl cyanide-4-phenylhydrazone (FCCP), the potassium uniporter valinomycin, or a combination of antimycin A and oligomycin A [79]. However, inherent cellular toxicity restricts their in vivo application. Recently, the anthelmintic drug niclosamide, approved by the FDA for the treatment of tapeworm infections, has been identified as an indirect PINK1 activator through the uncoupling of the mitochondrial membrane potential [80]. The potential therapeutic effect of niclosamide and its analogues has been reported in many diseases including Parkinson’s disease, cancer, type 2 diabetes, bacterial and viral infection, rheumatoid arthritis, and systemic sclerosis through multiple mechanisms such as uncoupling of oxidative phosphorylation, and modulation of Wnt/β-catenin, mTORC1, STAT3, NF-κB, and Notch signaling pathways [81,82]. Moreover, niclosamide and its analogues have been shown to exert renoprotective effects in various kidney models including unilateral ureteral obstruction, renal ischemia/reperfusion injury, adriamycin nephropathy, as well as db/db and STZ-induced type 1 diabetic kidney injury [83,84,85,86,87]. Despite its promising antifibrotic effect in kidney disease, the underlying mechanism is still not clear. To date, there is no study demonstrating the direct association of niclosamide with PINK1 in the kidney. Amplifying PINK1 activity and initiating PINK1 dependent mitophagy may well be a key mechanism of niclosamide in protecting against kidney injury, which deserves validation. Given the well-documented safety profile in vivo, niclosamide and its analogues may be repurposed as potent agents to treat DKD.

## 5. Conclusions and Perspectives

The involvement of PINK1 in regulated mitochondrial function, especially PINK1-mediated mitophagy, has been intensively studied in diabetic kidney disease. Although the function and activity of PINK1 and its downstream substrates are not fully elucidated, it is clear that PINK1 kinase plays key roles in maintaining mitochondrial function and cellular homeostasis in addition to regulating mitophagy. Recently, there is an increasing number of clinical trials with various therapeutic modalities by targeting different aspects of mitochondrial dysfunction, which include modulation of oxidative stress, augmentation of mitochondrial biogenesis, and stimulating mitochondrial dynamics [88,89]. Given the diverse roles of PINK1 in mitochondria, future studies to determine the association between PINK1 and the approved/ongoing clinical trial drugs such as Sonlicromanol (KH176), Bezafibrate, and NAD^+^ precursors/modulators deserve further exploration [88,89]. These studies will not only provide a deeper understanding of the molecular mechanism of the drugs but also identify novel PINK1-targeted agents for future clinical application. It is becoming increasingly recognized that defective PINK1-mediated mitophagy contributes to the pathogenesis of DKD. Therefore, pharmacologically activating PINK1-dependent mitophagy may be a promising therapeutic strategy for the treatment of DKD by enhancing the removal of damaged mitochondria. Future research to better understand the molecular basis for the protective role of PINK1 in DKD is likely to be therapeutically beneficial.

## Figures and Tables

**Table 1:** Functions of PINK1 with its putative substrates in healthy and damaged mitochondria.

Functions	Putative Substrates	Location of Putative Substrates	Phosphorylation Sites	References
**In Healthy Mitochondria**				
Anti-apoptosis	TRAP1	IMS/MIM	?	[23]
	HtrA2	IMS	Ser142	[24]
Regulation of mitochondrial respiration	NdufA10	MIM	Ser250	[25]
Regulation of cell growth or differentiation	mTORC2/Akt	Cytosol	?	[26]
	PKA/p47	Cytosol	?	[27,29]
Suppression of autophagy/mitophagy	Drp1	Cytosol	?	[28]
	PKA/LC3	Cytosol	?	[29]
	Parkin, DJ-1	Cytosol	/	[31]
Activation of autophagy	SQSTM1/P62	Cytosol	Ser28	[32]
**In Damaged Mitochondria**				
Activation of mitophagy	PINK1	MOM	Ser228, Ser402, Thr257, Thr313	[33]
	Parkin	Cytosol/MOM	Ser65	[35]
	Ubiquitin	Cytosol/MOM	Ser65	[36]
	E3 ubiquitin ligases such as SIAH1, ARIH1, and MUL1	MOM	/	[37,38,39]
	Mitofusin2	MOM	Thr111, Ser 442	[48]
	Rab8A	Cytosol	Ser111	[56]
Regulation of mitochondrial fission/fusion	Drp1	MOM	Ser616	[44]
	Mfn2	MOM	Ser378	[47]
Regulation of biogenesis	PARIS	Cytosol	Ser322, Ser 613	[51]
Regulation of mitochondrial motility	Miro	MOM	Ser156, T298/299	[53,54]
MDVs	syntaxin-17	MOM	/	[58]
Activation of autophagy	Beclin 1	Cytosol	/	[59]
Regulation of Ca^2+^ signalling	Na^+^/Ca^2+^ exchanger	MIM	/	[60]
	LETM1	MIM	Thr192	[61]
Anti-apoptosis	Bcl-xL	Cytosol	Ser 62	[62]

MOM, mitochondrial outer membrane, IMS, intermembrane space, MIM, mitochondrial inner membrane.

**Table 2:** Role of PINK1 in diabetic kidney disease.

Cell Type	Experimental Models	Animals’ Age or Week after Induction of Diabetes	PINK1 Expression Level	Phenotype	Role of PINK1	Ref.
**PTECs**	Renal proximal tubular epithelial cell lines HK-2 (human) and LLC-PK1 (porcine), STZ-induced diabetic mice	2 months	Decreased	The expression of PINK1, punctate LC3 and Mfn2 were decreased while the expression of Drp1 and Fis1 were upregulated, in the tubular of STZ-induced diabetic mice, which were normalized by inhibition of MIOX by D-glucarate.	Mitophagy	[63]
	Renal proximal tubular epithelial cell lines HK-2 (human), db/db mice	24 weeks of age	Decreased	The reduced expression of PINK, Parkin, LC3 II, Mfn2 and increased expression of Drp1 were found in the kidney of db/db mice, which were reversed by MitoQ.	Mitophagy	[64]
	Streptozotocin-induced diabetic rat	4 weeks	Increased	The increase expression in PINK1 and Drp1 were found in STZ-treated rats, which was associated with the loss of renal calpain 10.	Mitophagy	[65]
	db/db mice	21 weeks old	Increased	The expression of PINK1, Parkin, LC-3II, Drp-1, Fis-1, and MFF were increased in the kidneys of db/db mice, which were ameliorated by Astragaloside IV.	Mitophagy	[66]
	db/db mice	12 weeks	Increased	The expression of PINK1, Parkin, and Drp1 were upregulated in the db/db mice, which were reduced after Huangqi-Danshen decoction treatment.	Mitophagy	[67]
**Podocytes**	Mouse podocyte cell line, STZ-induced type 1 diabetes	8 weeks	Decreased	The decreased expression of PINK1 and increased apoptosis were found in HG treated podocytes and STZ induced mice, which were reversed by overexpression of FoxO1.	Mitophagy	[68]
	A mouse podocyte cell line (CIMPs), STZ-induced type 1 diabetes	12 weeks	Decreased	The expression of PINK1, Parkin, LC3I/II, and Mfn1 were decreased while the expression of Drp-1 and p62 were increased in HG-treated CIMPs and STZ-induced diabetic mice. However, these effects were reversed by FoxO1 overexpression.	Mitophagy	[69]
	Human renal biopsy samples, Human podocytes, STZ-induced type 1 diabetes	12 weeks	Decreased	The expression of PINK1, PARK2, PGC-1α and Sirt1 were decreased in HG-treated podocytes and in STZ induced diabetic mice, which were increased after rPGRN administration.	Mitophagy	[70]
	mouse podocyte cell line (MPC5), high fat diet (HFD)-induced obese rats	20 weeks of age	Increased	The expression of PINK1, Parkin, Beclin1, Atg5 and LC3 were increased PA treated podocytes and kidneys of HFD rats.	Mitophagy	[71]

## Data Availability

The data presented in this study are available on request from the corresponding author.
